# Renal cortical necrosis – a rare manifestation of dengue fever: A case report

**DOI:** 10.1097/MD.0000000000035719

**Published:** 2023-10-27

**Authors:** Fazal ur Rehman, Syeda Tayyaba Rehan, Fatima Yousaf, Navin Rathore, Bakhtawar Jamal Rind, Mohammed Mahmmoud Fadelallah Eljack, Muhammad Sohaib Asghar, Farruk Omair

**Affiliations:** a Department of Medicine, Aga Khan University Hospital, Karachi, Pakistan; b Dow University of Health Sciences, Karachi, Pakistan; c Liaquat National Hospital and Medical College, Karachi, Pakistan; d Jinnah Sindh Medical University, Karachi, Pakistan; e University of Bakhtalruda Faculty of Medicine and Health Sciences, AL-Dewaym, Sudan; f Department of Internal Medicine at Sun ’N Lake, AdventHealth, Sebring, FL; g Division of Nephrology and Hypertension, Mayo Clinic, Rochester, MN; h King Fahad Armed Forces Hospital, Jeddah, Kingdom of Saudi Arabia.

**Keywords:** anuria, case report, dengue fever, renal cortical necrosis, viral disease

## Abstract

**Rationale:**

Dengue fever is a widespread mosquito-borne viral disease, most prevalent in the tropical and subtropical areas of the world. There has been a significant rise in the incidence and number of outbreaks of dengue in recent years, which has made it a matter of global concern. It may be associated with a number of renal complications, ranging from hematuria, proteinuria, glomerulonephritis, and acute tubular necrosis. However, renal cortical necrosis (RCN) is a rare renal complication of this disease.

**Patients concerns:**

We report the case of a young gentleman who presented with fever, vomiting, and anuria. On workup, he was found to be having complicated Dengue fever with RCN resulting in acute renal failure.

**Diagnosis:**

To the best of our knowledge, RCN is not a reported renal complication of dengue fever.

**Interventions and outcomes:**

Our report highlights the importance of early consideration of renal cortical necrosis in patients with dengue fever and persistent anuria.

**Lesson:**

This would allow for better disease prognostication while enabling physicians to develop more effective treatment strategies.

**Take home messages**

- Acute febrile illness leading to acute renal failure in the absence of shock should raise the suspicion of uncommon causes of renal failure like RCN.
- When a patient presents with abdominal pain and acute renal failure, RCN should be one of the differential diagnoses.
- In unexplained Acute renal failure besides routine tests, CT/MRI, or renal biopsy should be considered to look for uncommon yet completely different causes of renal failure like RCN, in which a patient has to be counseled for long term hemodialysis or early renal transplant.

## 1. Introduction

Dengue is a common mosquito-borne viral disease, transmitted by the female species of Aedes Aegypti, which majorly thrives in the tropical and subtropical areas of the world.^[1]^ It has become a cause of serious global concern as recent years have seen a dramatic increase in the overall incidence as well as in the number of outbreaks of dengue. It is estimated that dengue is prevalent in over 125 countries and causes an estimated 100 million symptomatic infections with approximately 10,000 deaths per year.^[2,3]^ In Pakistan, from 1995 to 2019, there were around 147,200 cases of dengue infection and over 800 deaths recorded.^[4]^ We recently saw a massive dengue outbreak in Pakistan, beginning in July 2019, with a total of 48,906 reported cases, “between July and November 2019.”^[5]^

Dengue presents with a wide variety of clinical manifestations, ranging from a mild flu-like illness to life-threatening hemorrhagic fever and shock syndrome. It can also affect various organ systems including renal, neurological, hepatic, respiratory, cardiac, and gastrointestinal systems.^[6,7]^ Amongst the renal manifestations of dengue fever, a wide spectrum of disorders ranging from hematuria, proteinuria, glomerulonephritis, and acute tubular necrosis have all been described.^[8]^ However, we report a unique case of a patient who presented with dengue fever and anuria, which was found to be secondary to renal cortical necrosis (RCN). To the best of our knowledge, RCN is a very rare renal manifestation of dengue fever.

## 2. Case presentation

A young gentleman, presented to the Emergency Department of Tertiary Care Hospital in November 2019 with complaints of fever, vomiting, and loose stools for 4 days along with anuria for 2 days. Fever was documented at 38.9 degrees Celsius, continuous in nature with chills and rigors. It was associated with watery loose stools, around 4 episodes per day, containing no blood. Vomiting occurred 5 to 6 times per day, non-projectile, non-bilious, post-meals, with only food contents associated with mild diffuse abdominal pain. There was no history of rash or bleeding from any site. Two days prior to arrival, he had complaints of nil urine output. He does not have any significant past medical history. He is a nonsmoker and does not use any reactionary substances and there was no history of any allergy.

On arrival at the emergency room, he had a blood pressure of 102/68 mm Hg, Pulse of 88/min, Respiratory rate of 20/min, oxygen saturation of 99% on room air, and temperature of 37.6 degrees Celsius. He was dehydrated but not pale, cyanosed, or icteric and there was no lymphadenopathy. Abdominal examination showed bilateral flank tenderness, the rest of the systemic examination was unremarkable. A workup for acute febrile illness was sent. The urine detail report was normal as was the x-ray chest, blood cultures were sent, and he was started on empirical antibiotics. Being in an endemic area for dengue and malaria and the presence of thrombocytopenia, dengue antigen along with blood for malarial parasite (MP) and rapid antigen test for malaria were sent. Diagnosis of dengue fever was made on the basis of a positive dengue antigen test. Malaria was ruled out as a cause of the presentation by a negative peripheral film as well as a negative immunochromatographic test for malaria. Amylase and lipase were found raised and as were urea and creatinine. He was managed along the lines of complicated dengue fever, pancreatitis, and acute kidney injury (AKI). He was given 5 liters of intravenous fluids with no improvement in urine output. A blood culture was sent at the time of arrival and all subsequent cultures sent during the hospital course showed no growth of any bacterial pathogen, so antibiotics were de-escalated.

**Figure 1.**
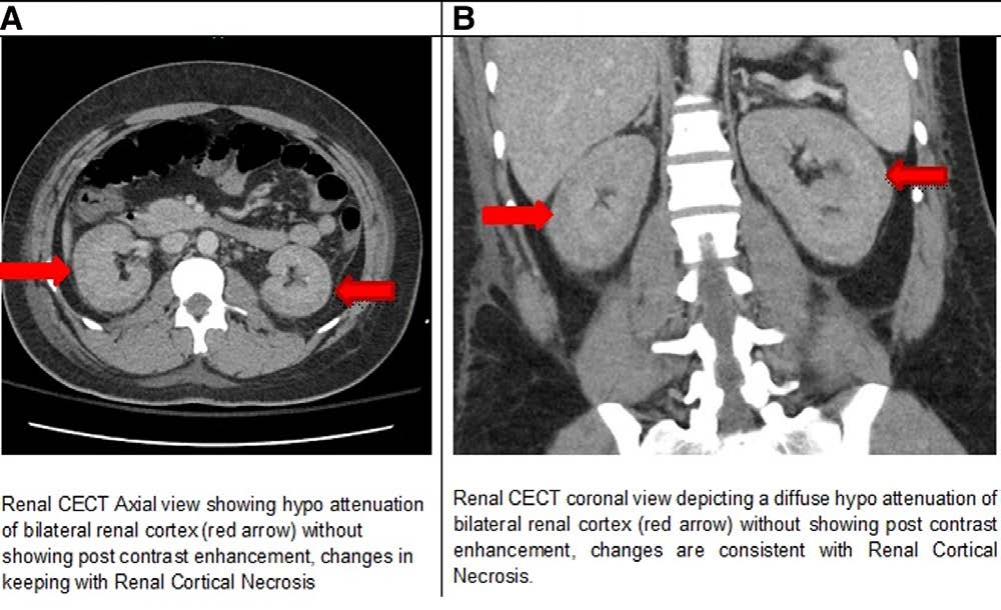
(A) Kidney contrast enhanced CT scan: axial section depicting renal cortical necrosis. (B) Coronal section depicting renal cortical necrosis.

He developed shortness of breath, on chest examination he had fine basal crepitation, and a repeat x-ray chest showed pulmonary edema as he was anuric so nephrology was taken on board and an urgent session of hemodialysis was done. Computed tomography (CT) of the Kidney, Ureter, and Bladder was done that reported the normal appearance of both kidneys and ureters, with no evidence of nephrolithiasis or obstructive nephropathy. However, there was minimal bilateral perinephric fat stranding noted. The impression was that of acute renal failure secondary to acute tubular necrosis though he never had any episode of hypotension. Further, sessions of hemodialysis were continued as needed. He continued to have a high-grade fever of up to 39 degrees Celsius and diffuse abdominal pain so CT scans abdomen with contrast were done to rule out any occult intra-abdominal collection which showed bilaterally swollen kidneys with perinephric fat stranding. No enhancement of renal cortex was identified on arterial and delayed phases of the scan with a slight enhancement of medullary regions, representing renal cortical necrosis as shown in Figure 1. Conservative treatment was continued, his platelet counts were also serially followed and they showed an improving trend. He was discharged after 12 days of hospital stay and advised thrice weekly hemodialysis. His creatinine and blood urea nitrogen levels continued to be elevated throughout the hospital stay and were 9.9 and 58 mg/dL, respectively at the time of discharge along with persistent anuria.

His initial laboratory investigations are presented in Table 1. The autoimmune workup and coagulation profile was negative. Blood and urine cultures didn’t grow any organisms. CT scan abdomen showed RCN.

**Table 1:** Laboratory investigations on admission.

Investigation	Results
Blood urea nitrogen	37.48 mmol/L
Serum creatinine	1432 µmol/l
Serum potassium	3.4 mmol/L
Serum bicarbonate	19.6 mmol/L
Hemoglobin	104 g/L
Mean corpuscular volume	88 fl
Leukocyte	12.1 × 10^9^/L
Platelet count	72 × 10^9^/L
Peripheral blood film	Anisocytosis, mild polychromasia platelets low on film, monocytosis, and no fragmented red blood cells
Lactate dehydrogenase	31.85 µkat/L
Total bilirubin	17.10 µmol with 8.5 µmol direct bilirubin and 8.5 µmol indirect bilirubin
Gamma glutamyltransferase	4.36 µkat/L
Alanine aminotransferase	2.29 µkat/L
Alkaline phosphatase	0.75 µkat/L
Aspartate transaminase	1.49 kat/L
Serum lipase and amylase	6.95 µkat/L and 6.15 µkat/L, respectively

## 3. Differential diagnosis

The differential diagnosis, in this case, can be hemolytic uremic syndrome (HUS), complicated malaria, or sepsis secondary to pyelonephritis. HUS was rolled out by normal peripheral film with no schistocytes on repeated occasions. MP and MP immunochromatographic test were negative for malaria and sepsis was ruled out by negative cultures, fever subsided after antibiotics were stopped and there was no source of sepsis.

## 4. Treatment

Initially treated with oxygen via nasal cannula of up to 2 liters, intravenous fluid, and empirical antibiotics later on needed Hemodialysis for symptoms of uremia and pulmonary edema. All cutlers were negative so antibiotics were discontinued. The fever subsided on day 6th of admission along with improvement in platelet count but renal function didn’t get better.

## 5. Outcome and follow up

He was discharged with the decision to thrice weekly hemodialysis as he remains anuric.

The last follow-up was 1 month after discharge on December 21st, 2019 in the nephrology clinic, still anuric, and advised maintenance dialysis and regular follow-up in the clinic to decide further treatment options.

## 6. Discussion

Dengue fever has been associated with a variety of renal disorders, ranging from proteinuria, hematuria, glomerulonephritis to renal tubular necrosis.^[8]^ Studies have reported the incidence of AKI in dengue to be between 10.8 to 14.51%.^[9–11]^ Several mechanisms have been proposed to elucidate the pathogenesis involved in the renal manifestations of dengue fever. Amongst the processes described are a direct invasion of renal parenchyma, deposition of immune complexes in glomeruli, HUS, and rhabdomyolysis.^[8,11–14]^ Dengue may also cause leakage of intravascular fluid, resulting in shock and decreased renal perfusion, leading to acute tubular necrosis.^[9,11]^

RCN is a very rare form of AKI, with an incidence of less than 2% in developed countries and 6% to 7% in developing countries.^[15]^ Its incidence in dengue fever is very exceptional. To the best of our knowledge there is a single published case report on RCN in dengue fever documented by Mazumdar.^[16]^ The clinical presentation was the same as in our patient with a short history of fever, abdominal pain anuria, and with no shock but in their case, the diagnosis was confirmed by the gold slandered test of renal biopsy after CT scan finding suggestive of RCN, although the outcome was the same as both patients needed maintenance dialysis.^[16]^

RCN has been noted as a complication of other tropical infections like vivax malaria.^[17]^ It has potentially serious and adverse outcomes. In the complete variety of RCN, there is a complete loss of kidney function and irreversible end-stage renal disease, while in the incomplete form of this disease, there might be some recovery of the renal tissue, depending on the extent of necrosis.^[15]^ The exact mechanism of RCN is poorly understood. However, renal hypoperfusion and vascular endothelial injury have been widely established underlying mechanisms. Both renal ischemia and endothelial injury cause endothelin release from vascular endothelial cells, resulting in renal damage.^[15,18]^ The most common presenting features of this condition are anuria along with raised serum creatinine levels,^[15,19]^ which were also evident in our patient. The gold standard for diagnosing RCN is a renal biopsy, which will demonstrate ischemic necrosis of all renal cortex components (glomeruli, blood vessels, and tubules).^[15,20]^ However, contrast enhanced CT scan and Magnetic resonance imaging (MRI) can be used as alternative modalities to reliably diagnose RCN.^[21,22]^ Characteristic CT scan findings include the absence of the renal cortex enhancement, along with enhancement of the renal medulla and the absence of renal excretion.^[21]^ CT scan imaging done on our prototype reported similar findings, hence concurrent with the diagnosis of RCN.

## Author contributions

**Conceptualization:** Fazal ur Rehman.

**Data curation:** Syeda Tayyaba Rehan, Navin Rathore.

**Investigation:** Bakhtawar Jamal Rind.

**Project administration:** Farruk Omair.

**Resources:** Farruk Omair.

**Software:** Muhammad Sohaib Asghar.

**Supervision:** Muhammad Sohaib Asghar.

**Writing – original draft:** Fatima Yousaf.

**Writing – review & editing:** Mohammed Mahmmoud Fadelallah Eljack, Muhammad Sohaib Asghar.
